# Exploring Microbiota Diversity in Cervical Lesion Progression and HPV Infection through 16S rRNA Gene Metagenomic Sequencing

**DOI:** 10.3390/jcm12154979

**Published:** 2023-07-28

**Authors:** Irina Livia Stoian, Anca Botezatu, Alina Fudulu, Ciprian Gavrila Ilea, Demetra Gabriela Socolov

**Affiliations:** 1 Department of Obstetrics and Gynecology, ‘Grigore T. Popa’ University of Medicine and Pharmacy, 700115 Iasi, Romania; stoian.irinalv@yahoo.com (I.L.S.); demetrasocolov@gmail.com (D.G.S.); 2 Stefan S. Nicolau Institute of Virology, Romanian Academy, 030304 Bucharest, Romania

**Keywords:** cervicovaginal microbiota, HPV infection, metagenomics, cervical cancer, next-generation sequencing

## Abstract

(1) Background: Cervical cancer is a significant health concern, with the main cause being persistent infection with high-risk Human Papillomavirus (hrHPV). There is still no evidence for why viral persistence occurs in some women, but recent studies have revealed the interplay between cervical microbiota and hrHPV. This research aimed to characterize the cervicovaginal microbiota in cervical lesion progression and HPV infection status. (2) Methods: This study included 85 cervical specimens from women from the north-eastern region of Romania. DNA was isolated from cervical secretion for HPV genotyping and 16S ribosomal RNA gene NGS sequencing. (3) Results: Our study revealed a distinct pattern within the studied group when considering Lactobacillus species, which differs from findings reported in other populations. Specifically, the presence of *Lactobacillus iners* coupled with the absence of *Lactobacillus crispatus* alongside *Atopobium* spp., *Prevotella* spp., and *Gardnerella* spp. could serve as defining factors for severe cervical lesions. The results also showed a significant association between microbiota diversity, HPV infection, and cervical lesion progression. (4) Conclusions: As the microbiota profile seems to vary among different populations and individuals, a deeper comprehension of its composition has the potential to develop personalized detection and treatment approaches for cervical dysplasia and cancer.

## 1. Introduction

Cervical cancer is a significant global health issue, ranking as the fourth most diagnosed cancer among women. In 2020, it was estimated that there would be approximately 604,000 new cases of cervical cancer diagnosed and 342,000 deaths worldwide due to this disease. These numbers highlight the urgent need for effective prevention, early detection, and treatment strategies to reduce the burden of this pathology on women’s health globally [1].

In Romania, despite being a preventable disease, cervical cancer ranks as the third most prevalent cancer among women, following breast and colorectal cancer [2]. Based on data from the World Health Organization (WHO), Romania has the highest mortality rate due to cervical cancer in the European Union (EU), with approximately 10.84 deaths per 100,000 cases. Romania’s high rate of cervical cancer mortality is comparable with certain countries in South America, such as Peru (11.29 deaths per 100,000 cases) and Ecuador (9.95 deaths per 100,000 cases) [3].

The primary cause of cervical intraepithelial neoplasia (CIN) and cervical carcinoma is persistent infection with high-risk Human Papillomavirus (hrHPV), HPV16 being the most prevalent and significant genotype [4]. There is still no evidence for why viral persistence occurs in some women and not in others, but some recent studies have revealed the interplay between cervical microbiota, which modulates the immune status of the female genital tract, and viral infection [5].

The cervical microbiota is represented by the various microorganisms that colonize the reproductive tract. In a healthy cervical microbiota, *Lactobacillus* species such as *Lactobacillus crispatus*, *Lactobacillus jensenii*, *Lactobacillus gasseri*, and *Lactobacillus iners* dominate the microbial community. These *Lactobacillus* species contribute to a well-balanced environment with a low pH, creating a protective barrier against viral and bacterial pathogens. In contrast, an increased risk of infection has been associated with the presence of anaerobic pathogens, including *Gardnerella*, *Atopobium*, and *Dialister* [6]. However, *Lactobacillus iners* has been reported to dominate the microbial community when the vaginal microbiota transitions between normal and abnormal states and indicates susceptibility to CIN, which is also correlated with a higher frequency of HIV, HPV, and HSV-2 [7].

In a study from 2011, Ravel et al. classified the cervicovaginal microbiota into five community state types (CSTs). These CSTs were determined based on the presence and relative abundance of species identified through 16S rRNA gene sequencing. *Lactobacillus* spp. are the most common bacteria in CSTs I, II, III, and V, while anaerobic genera like *Prevotella, Atopobium, Dialister, Gardnerella, Aerococcus, Sneathia, Eggerthella, Finegoldia, Megasphaera, Peptoniphilus*, and *Mobiluncus* are more common in CST IV [7,8].

Recent studies have indicated that the cervicovaginal microbiome plays a crucial role in HPV infection persistence and the subsequent development of cervical precancerous or cancerous lesions [6,9]. As the diversity of the microbiota increases, so does the severity of cervical lesions and the persistence of the virus. Several potential mechanisms have been suggested, including reduced production of protective lactic acid, H_2_O_2_, and bacteriocin by *Lactobacillus*, disruption of mucosal integrity facilitating viral entry, and higher levels of oxidative stress induced by dysbiosis [9].

Among the four predominant *Lactobacillus* species, *L. crispatus* and *L. iners* both produce lactic acid, but they exhibit notable differences, especially concerning their impact on host health. The presence of *L. crispatus* in the vaginal environment has consistently been linked to good health, whereas communities dominated by *L. iners* may offer the host fewer protective benefits [10,11]. Importantly, it should be noted that *L. iners* frequently coexists with various bacterial species that colonize the vagina during instances of bacterial vaginosis [10,12]. Additionally, vaginal microbiota dominated by *L. iners* tends to have a higher vaginal pH compared to that dominated by *L. crispatus* [7]. A recent study indicates that the population of *L. iners* tends to increase in women who are HPV-positive and in those with LGSIL and HGSIL cytology. This particular increase is associated with a greater likelihood of transitioning to a dysbiotic vaginal microbiota. Furthermore, *Lactobacillus iners* has been linked to a higher risk of developing cervical dysplasia [13].

Some species, such as *Sneathia* spp., may also help HPV to enter and stay in the body by affecting cellular targets, such as the expression of immunosuppressing cytokines.

Hence, considering the vaginal microbiome as a promising marker not only for HPV infection but also for cervical precancerous lesions holds significant importance [5]. Women diagnosed with High-Grade Squamous Intraepithelial Lesions (HGSILs) exhibited notably higher levels of *Sneathia sanguinegens*, *Anaerococcus tetradius*, and *Peptostreptococcus anaerobius* and a lower abundance of *Lactobacillus jensenii* in comparison to women diagnosed with Low-Grade Squamous Intraepithelial Lesions (LGSILs) [14].

HPV, as a pathogenic factor, interacts with the cervicovaginal microbiome, resulting in the proliferation of abnormal microbiota. Conversely, it is also possible that the presence of abnormal microbiota increases susceptibility to HPV acquisition. Moreover, the dysbiosis of the cervical microbiota has been found to be associated with HPV infection and its persistence [14].

Based on what we know so far, there may be a link between HPV that does not go away and changes in the microbiome. For example, 11% of women with persistent HPV have bacterial vaginosis, compared to only 5% of women whose HPV infection clears itself [15]. Moreover, vaginal microbial dysbiosis plays a significant role in driving genital inflammation, so women with a microbiota dominated by *Gardnerella* spp. or *Prevotella* spp. had the highest genital inflammatory profiles [16,17].

Recently, it was shown that abnormal vaginal microbiota plays a significant role in the development of cervical cancer. In the lower female reproductive tract, a healthy or normal microbiota is characterized by low microbial diversity, with one or a few species of Lactobacillus dominating the microbial community, whereas a higher diversity was found in HPV-infected women [18,19].

Moreover, Brotman et al. conducted a study that revealed a potential association between specific types of vaginal microbiota and the clearance or persistence of HPV infection [18].

Numerous factors, such as ethnicity, way of life, location, and prior infectious history, can affect the cervical microbiota’s composition [20,21].

Therefore, it is crucial to characterize the diversity and composition of the microbiota in different individuals, particularly to understand its role in cervical cancer progression and persistent HPV infection, the latter being the leading risk factor in oncogenesis.

Taking together these data, the main objective of this study was to investigate the role of the cervicovaginal microbiota in the progression of cervical lesions associated with HPV infection in women from the north-east region of Romania. Considering recent findings, our specific objective was to examine the presence of *Lactobacillus crispatus* in women with normal cytology.

Additionally, we aimed to explore whether *Lactobacillus iners* could be associated with low-grade cervical dysplasia (LGSIL) or *Sneathia* with high-grade cervical dysplasia (HGSIL). The presence of these three microorganisms may allow us to establish an affordable PCR test in order to improve cytological and HPV screening. Moreover, this study seeks to identify microbiological markers that may be linked to the severity of cervical lesions and HPV infection and that may be added to the actual screening strategy (Pap and HPV tests). Furthermore, we planned to evaluate the functional composition of the microbiome in order to gain a comprehensive understanding of its role in these conditions.

## 2. Materials and Methods

### 2.1. Study Group and Design

This study included 85 cervical specimens that fit the inclusion criteria from a total of 345 women who self-referred for gynecological examinations in “Cuza Voda” Clinical Hospital of Obstetrics and Gynecology between April 2021 and June 2022. The inclusion criteria for this study were as follows: women aged 18 and above, not currently pregnant, who had not taken any antibiotics, probiotics, or immunosuppression medication for at least one month prior to sampling. Additionally, participants were required to have abstained from vaginal contact or showers for a minimum of three days prior to sampling. Prior to inclusion in this study, all participants were fully informed about the purpose and details of the research and provided their informed consent by signing a consent form.

The patients were invited to fill out a standardized questionnaire designed to gather information regarding their ethnicity, education, age, marital status, smoking and drinking habits, medical history, sexual activity, and place of residence.

During this study, all patients underwent a single visit, and samples for all assays were collected at that specific time. This method makes sure that all data points and samples are collected at the same time. This reduces the chance of differences from multiple visits and allows for a more accurate analysis of the role of the cervicovaginal microbiota in the progression of cervical lesions and HPV infection in the participants.

For microbiota, the cervical secretion was collected and preserved using an ESwab (COPAN-Brescia, Italy), a combination of a flocked swab and 1 mL of Liquid Amies. The specimens were stored at −80 °C until they were utilized. For the Papanicolaou test and HPV genotyping analysis, a cervical sample was collected using a separate Cervex Brush. The liquid-based preparation method for collecting cervicovaginal samples was performed according to the manufacturer’s instructions (ThinPrep-Hologic, Bedford, MA, USA), and the samples were stored until further analysis.

**Figure 1:**
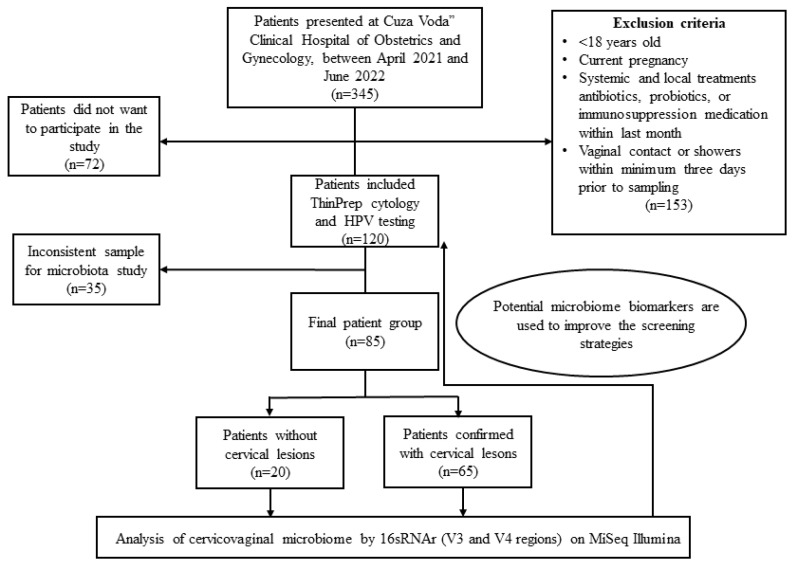
Flow diagram of this study.

Diagnoses were made following the grading criteria of the Bethesda System, which includes the following categories: Negative for Intraepithelial Lesion or Malignancy (NILM); Atypical Squamous Cells of Undetermined Significance (ASCUS), Atypical Squamous Cells That Cannot Exclude High-Grade Squamous Intraepithelial Lesion (ASCH); Low-Grade Squamous Intraepithelial Lesion (LGSIL); High-Grade Squamous Intraepithelial Lesion (HGSIL); and Squamous Cell Carcinoma (SCC). This criterion qualified 120 women for inclusion in this study (Figure 1).

### 2.2. DNA Isolation

DNA was isolated from cervical samples using a PureLink™ Microbiome DNA Purification Kit (Invitrogen, Waltham, MA, USA), according to manufacturer’s recommendations. Isolated DNAs were subsequently stored at −20 °C. The concentration and purity of each DNA sample were evaluated with a NanoDrop ND-1000 spectrophotometer (Thermo Fisher Scientific Inc., Waltham, MA, USA).

### 2.3. HPV Detection and Genotyping

Human Papillomavirus detection and genotyping was performed for all samples using INNO-LiPA^®^ HPV Genotyping Extra II (Fujirebio Europe, Ghent, Belgium) according to the manufacturer’s instructions. The kit contains 32 HPV-genotype-specific probes, SPF10 primer set, and human DNA control primers and provides ready-to-use amplification reagent HPV genotypes. The method allows the stratification of samples into high-risk (hrHPV), low-risk (lrHPV), and undetermined risk HPV types. The test includes 13 high-risk types: 16, 18, 31, 33, 35, 39, 45, 51, 52, 56, 58, 59, and 68.

### 2.4. 16S rRNA Gene Sequencing

For sequencing the variable V3 and V4 regions of the 16S rRNA gene, the 16S Metagenomic Sequencing Library kit from Illumina (San Diego, CA, USA) was utilized. The sequencing protocol was specifically designed to analyze the prokaryotic 16S ribosomal RNA gene (16S rRNA), and it was carried out following the manufacturer’s recommendations. For this, gene-specific primers that target the 16S V3 and V4 regions with overhang adapters attached were used (F:5′-TCGTCGGCAGCGTCAGATGTGTATAAGAGACAGCCTACGGGNGGCWGCAG-3′, R: 5′-GTCTCGTGGGCTCGGAGATGTGTATAAGAGACAGGACTACHVGGGTATCTAATCC-3′) [22].

For each sample, a mix was prepared that contained 12.5 µL 2X KAPA HiFi HotStart ReadyMix (Roche Molecular Systems, Inc.: Pleasanton, CA, USA), 5 µL (1 μM) Forward Primer, 5 µL (1 μM) Reverse Primer, and 2.5 µL (5 ng/µL) DNA sample to a final volume of 25 µL. PCR conditions were as follows: initial denaturation at 95 °C for 3 min; 25 cycles of 95 °C for 30 s, 55 °C for 30 s, and 72 °C for 30 s; final extension at 72 °C for 5 min. The amplification was followed by a PCR Clean-Up step that purified the 16S V3 and V4 amplicon by removing free primers and primer dimer species. Briefly, every sample obtained was mixed with 20 µL AMPure XP beads and shaken at 1800 rpm for 2 min. The plate was placed on the magnetic stand, and the supernatant was removed. Next, the beads were washed 2 times with 200 µL freshly prepared 80% ethanol. After the beads air-dried, a volume of 52.5 µL 10 mM Tris (pH = 8.5) was used for resuspension. The plate was placed on a magnetic stand, and a volume of 50 µL of supernatant was transferred to a new plate.

### 2.5. Library Preparation

The purified amplicons were subjected to an Index PCR step that allows for the labelling of the samples by attaching dual indices and adapters. For this, 5 µL of sample was mixed with 25 µL of 2X KAPA HiFi HotStart ReadyMix, 5µL of Nextera XT Index 1 Primers (i5), 5 µL Nextera XT Index 2 Primers (i7), and 10 µL of PCR-grade water. PCR conditions were as follows: initial denaturation at 95 °C for 3 min; 8 cycles of 95 °C for 30 s, 55 °C for 30 s, and 72 °C for 30 s; final extension at 72 °C for 5 min. This was followed by a new cleaning step of the final library with ethanol 80%. The final step included libraries quantification using a Qubit 2.0 Fluorometer (Thermo Fisher Scientific, Waltham, MA, USA). All libraries were pooled in equal molar ratios and denaturated with 0.2 N NaOH for sequencing. Samples were sequenced using an Illumina MiSeq v2 600 cycle kit (Illumina, San Diego, CA, USA).

### 2.6. Sequencing Data Analysis/Bioinformatics Analysis

Data were analyzed using the EasyMap pipeline (available for public use at https://yassour.rcs.huji.ac.il/easymap, accessed on 27 January 2023). This tool was developed using the shiny R package (version 1.6.0), along with the MaAsLin2 R package (version 1.4.0) for multivariate linear regression [23]. EasyMap integrates different pipelines, such as Quantitative Insights Into Microbial Ecology 2 (QIIME2, 2018.4.0 version), Linear Discriminant Analysis Effect Size (LefSe, 1.0.8.post1 version), and Phylogenetic Investigation of Communities by Reconstruction of Unobserved States (PICRUSt, 1.1.3 version), and incorporates the databases of Greengenes, SILVA, and UNITE [24,25,26,27].

Fastq.gz paired files generated by MiSeq sequencing were uploaded along with sample metadata (defines studied group for each sample) and manifest files (identifies sample libraries) to EasyMAP portal (http://easymap.cgm.ntu.edu.tw/, accessed on 27 January 2023) [22]. After the sequences were demultiplexed (q2-demux), q2-dada2 was used to trim low-quality reads (Q < 25), merge pair-end high-quality reads, and remove chimeric sequences. The following step was taxonomic analysis using q2-feature-classifier, and “Greengenes_V4_classifier” was used for microbiome function prediction. Frequency bar graphs for phyla and heatmaps for genera visualization were plotted in R and Excel (Microsoft Office 365). For alpha diversity analysis, EasyMAP generated observed operational taxonomic units (OTUs), Faith’s phylogenetic diversity, and Shannon index. A sequence clustering approach was employed, considering a 97% similarity threshold, where sequences with high similarity from the same taxa were grouped together into a single OTU. Statistics of alpha diversity analysis for all groups and pairwise Kruskal–Wallis tests (*p* < 0.05) were performed and visualized using box plots. Beta diversity analysis was assessed with PERMANOVA statistical analysis (*p* < 0.05) using unweighted unifrac and weighted unifrac, Bray–Curtis and Jaccard, respectively.

Principal coordinates analysis (PCoA) was used to visualize microorganism composition and abundance similarity/dissimilarity between samples. For statistical analyses, we categorized the data into two groups based on HPV infections and six groups based on cervical cytology results. This decision was made according to multiple clustering analyses since it has been confirmed that organisms clustered in the same group are related to similar diagnosis and HPV infection. Pearson’s correlation and PCoA mainly sustain the clustering by disease stage compared to HPV infection status.

Linear Discriminant Analysis Effect Size (LEfSe), a taxonomy differential abundance analysis, was used to identify microorganisms with significant differences between groups from KEGG level 1~level 3 and the Wilcoxon test (α < 0.05) [28]. Taxonomy differential abundance was considered significant only when the linear discriminant analysis (LDA) score was greater than 2. Taxa abundance was plotted using bar, heatmap, and circular cladogram graphics provided by the EasyMAP platform or plotted using R.

Furthermore, microbiome function related to HPV infection and in different cytological types was predicted by using taxa abundance differentially expressed and the PICRUSt, which mapped Greengenes IDs to the corresponding KEGG pathway. The predicted microbiome function was plotted by circular graphics using a log10 LDA score.

The heatmaps and hierarchical clustering (Euclidian distance) were performed using Morpheus Broad Institute matrix visualization and analysis software (https://software.broadinstitute.org/morpheus/, accessed on 15 February 2023) [29].

### 2.7. Statistical Analysis of Demographics Data

Statistical analysis was conducted with GraphPad Prism 6 software (GraphPad Software Inc., San Diego, CA, USA). The results were reported as medians, which allowed for the comparison of differences among the investigated groups. For qualitative variables, Chi-square and Fisher’s exact tests were applied, while the analysis of variance (ANOVA) was used for quantitative variables. Statistical significance was defined as a *p*-value of less than 0.05.

### 2.8. Ethics Statement

This study was conducted according to the principles expressed in the Declaration of Helsinki. It was approved by the University of Medicine and Pharmacy Grigore T. Popa Ethic Committee (no 57/17.03.2021). Written informed consent was obtained from all participants.

## 3. Results

### 3.1. Patient Cohort Sociodemographic and Characteristics

To characterize the cervical microbiota of subjects included in this study, we divided them into seven groups according to the Papanicolaou test results and considering the presence or absence of HPV, as follows: LGSIL (Low-Grade Squamous Intraepithelial Lesion) (*n* = 18, age range: 21–54 years old), HGSIL (*n* = 9, age range: 28–54 years old), ASCUS (Atypical Squamous Cells of Undetermined Significance) (*n* = 16, age range: 20–56 years old), ASCH (Atypical Squamous Cells) (*n* = 13, age range: 26–59 years old) and squamous cervical carcinomas (SCCs) (*n* = 9, age range: 38–87 years old). Also, an NILM (Negative for Intraepithelial Lesion or Malignancy) group (*n* = 20) comprising 11 HPV-negative samples (NILM-) (age range: 29–62 years old) and 9 HPV-positive samples (NILM+) (age range: 25–47 years old) was included. All patients with an abnormal Pap smear, current or previous HPV infection, cervical lesion suspicion, or symptoms (e.g., abnormal vaginal bleeding) underwent colposcopy. All cases with ASCUS, LGSIL, ASCH, HGSIL, and SCC underwent targeted biopsy for further evaluation. However, the biopsy results did not alter the classification for the mentioned cytological classes (Table S1).

There is a direct relationship between the Pap smear and the biopsy; thus, the anatomopathological result confirmed severe dysplasia and carcinomas. The results of mild dysplasia were also confirmed to be of low risk following the biopsy (16/18–88.8%). Therefore, we can be guided by the Pap test result regarding the severity of the cervical lesions in order to assess microbiota screening.

### 3.2. Relationships between Cytological Status and Demographic Characteristics in the Studied Groups

The data analysis revealed that age plays a significant role in the development of cancer. Our results showed that the SCC+ group had a higher median age of 63.20 ± 12.260 years. Additionally, significant associations were observed between educational level, living area, marital status, and sexual frequency. A higher frequency of cervical cancer cases was observed in rural areas compared to urban areas within the SCC group (Table 1).

### 3.3. Distribution of HPV Genotypes in Studied Groups

**Figure 2:**
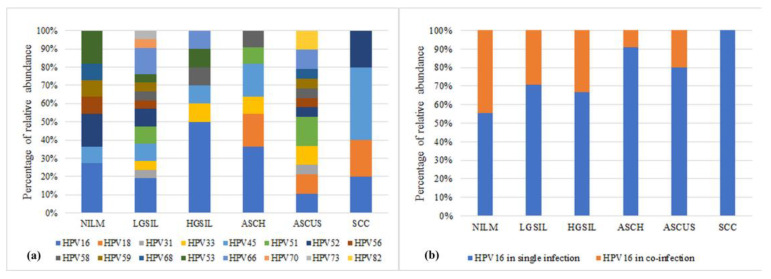
(**a**) Graphic representation of HPV genotype distribution. (**b**) HPV16 genotype distribution in studied groups.

All patients included in this study underwent HPV testing, and the results showed that 81.18% (69/85) of the samples tested positive for HPV. Among the NILM samples, 45% (9/20) presented HPV infection. The patients displayed a variety of HPV infections, with some individuals being infected with a single HPV genotype, while others exhibited multiple genotypes. The most prevalent high-risk genotypes were HPV16 (27.90%) and HPV45 (11.70%). Other frequent HPV genotypes found in the samples included HPV51, HPV52, and HPV66, each with a prevalence of 8.70%. In 21.74% of the samples, the specific HPV genotype could not be identified. Additionally, some samples exhibited HPV infection with low-risk genotypes. Among all HPV-positive samples, 62.35% (*n* = 53) had single HPV infections, while 18.82% (*n* = 16) had co-infections with multiple HPV genotypes (Figure 2a).

In terms of the diversity of HPV genotypes within the studied groups, it was found that the LGSIL and ASCUS groups exhibited the highest diversity (13 and 12 genotypes, respectively). On the other hand, the SCC group displayed the lowest diversity, with only four genotypes identified. Notably, the SCC group exclusively consisted of patients with single HPV infections, and the identified genotypes were HPV16, HPV18, HPV45, and HPV52. Furthermore, an analysis of the percentage of single infections with HPV16 revealed an increase, although not statistically significant, as the lesion progressed from NILM to SCC (Figure 2b).

### 3.4. Cervicovaginal Microbiota Diversity and Composition among Studied Groups

**Figure 3:**
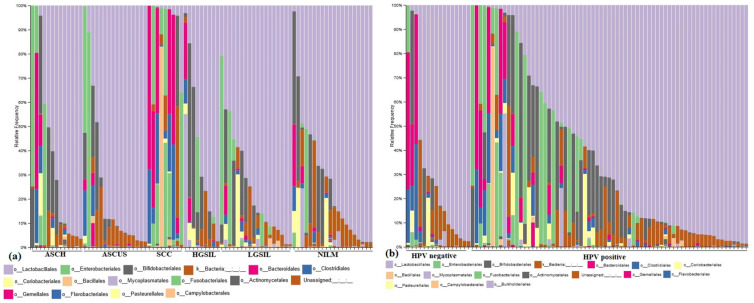
(**a**) The cervical microbiota of control, cervical dysplasia, and cervical cancer individuals with and without HPV. (**b**) Cervical microbiota diversity in HPV-positive and -negative samples.

The bacterial community composition varied between the investigated groups, each group presenting a characteristic pattern (Figure 3a). While the NILM group presents a high frequency of Lactobacillales order, the dysplasia individuals presented a decreased composition of *Lactobacillales* representative, a higher frequency of *Enterobacteriales* and *Bifidobacteriales* orders, and a particularly notable presence of *Gardnerella vaginalis*. The SCC group displayed a higher complexity of microbiota, with the most dominant orders being *Bacteroidales* (specifically *Prevotella* spp.), *Bifidobacteriales*, *Enterobacteriales,* and *Bacillales* (including *Staphyloccocus* spp.).

Across all groups, there was a lower frequency of the Coriobacteriales order (specifically *Atopobium* spp.). In the HPV-positive group, there was a higher diversity of microbiota, with the predominant orders being *Bifidobacteriales*, *Enterobacteriales*, *Bacteroidales*, and *Mycoplamatales* (Figure 3b). Among the HPV-positive individuals, 44% (30/69) exhibited a higher frequency of the *Lactobacillales* order.

**Figure 4:**
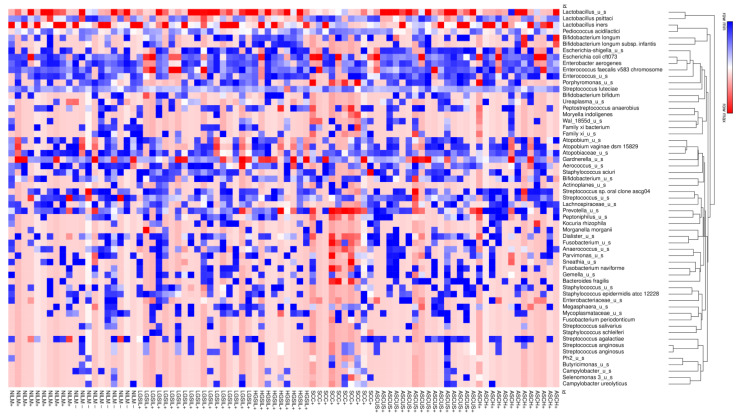
Heatmap of relative microbiome abundance found in vaginal samples obtained from women included in this study through 16S rRNA gene sequencing. Row hierarchy clustering was performed calculating the Euclidean distance based on hierarchical analysis to identify and order the most prominent taxa related to the microbiome profiles.

The heatmap analysis highlights the most important clusters of cervical microbiota at the species level. The analysis showed that *Lactobacillus_u_s* (*L. unclassified*) and *L. iners* were predominant species in cluster I, *Escherichia coli* along with *Enteroccocus* spp. Were predominant species in cluster II, and *Atopobium* spp. Was predominant in cluster III. The clusters IV and V are marked by *Prevotella* spp., *Streptoccocus* spp., and *Staphyloccocus* spp., and the most dominant species in cluster VI was represented by *Gardnerella* spp. (Figure 4).

Further, we compared the diversity of cervical microbiota among the studied groups at the genus and species levels. It was observed that the *Lactobacillus* genus was the most dominant in all studied groups, except the SCC group. The highest abundance of *Lactobacillus* was exhibited by the samples included the NILM- (67.56%) and ASCUS (66.01%) groups, with *L. iners* as the dominant species. In contrast, the SCC group displayed the lowest abundance of *Lactobacillus* (7.75%). The most prevalent *Lactobacillus* species within the investigated groups was *Lactobacillus iners*, even in the NILM- study group. The differences in abundance between the groups were statistically significant (*p* < 0.0001). The HPV-negative group demonstrated a higher prevalence of *Lactobacillus* compared to the SCC group, which exhibited the lowest prevalence.

*L. iners* displayed the highest abundance in the negative NILM group (42.84%), while *L. unclassified* emerged as the predominant species in the ASCUS group (41.97%). Certain species like *L. psittaci* or *L. zaea* showed lower levels of occurrence in the cervical samples, while others, such as *L. crispatus*, were not detected at all.

**Figure 5:**
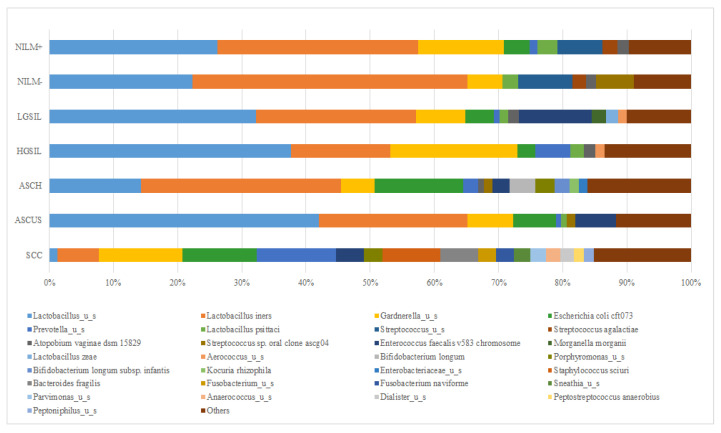
Taxa relative abundance (%) for cervical samples with different cytology groups and related to the HPV infection (positive +, negative −).

*Gardnerella_u_s* was predominantly detected in women with HGSIL (19.78%), followed by women with positive NILM results (13.43%) and SCC (12.98%) cases (Figure 5). *Prevotella_u_s* was primarily identified in SCC-positive samples (12.36%) and HGSIL samples (5.45%), while its presence in other groups was nearly negligible. Additionally, other species were also identified: *Escherichia coli cft*073, which was found in ASCH and SCC samples (13.80% and 11.62%, respectively), and *Enterococcus faecalis v*583 *chromosome*, which was detected in women with LGSIL (11.34%) and ASCUS (6.36%) results.

The taxonomic composition varies significantly (*p* < 0.0001) between HPV-positive samples based on the presence of a single genotype or co-infection status. One of the most important observations is that *L. iners* is more abundant in individuals with a single HPV genotype, whereas samples with co-infection are characterized by the presence of *Lactobacilus_u_s* in association with a high abundance of *Prevotella _u_s.*

**Figure 6:**
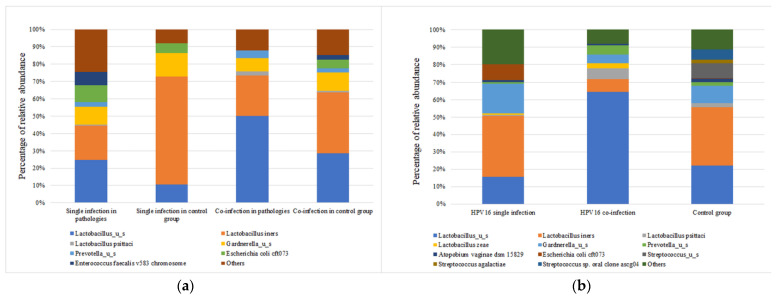
(**a**) Taxa relative abundance (%) in individuals with a pathological (abnormal cytology result) and control group related to HPV infection: single or co-infection. (**b**) Taxa relative abundance (%) in individuals with HPV16 infection (single or co-infection).

Because HPV16 is the most prevalent genotype in our study group, we evaluated the abundance of most important taxa with HPV16 in single and co-infection individuals (Figure 6a). The mean abundance of taxa differs significantly (*p* < 0.0001) between the two categories. As can be observed, *Lactobacillus_u_*s is more abundant in the HPV16 co-infection group, while *L. iners* more abundant in the single-infection group. Individuals with HPV16 co-infection show lower abundances of *L. psittaci* and *L. zeae*. Moreover, the HPV16 co-infection group has a slightly increased abundance of *Prevotella_u_s*, while the HPV16 single group showed an increased abundance of *Gardnerella_u_s* and *Escherichia coli cft*073 (Figure 6b).

### 3.5. Comparison of Samples’ Diversities

Alpha diversity was assessed using Shannon, Faith’s, and OTU diversity analyses. The Shannon index revealed significantly higher microbial diversity in the HGSIL and SCC groups (*p* = 0.0219, Kruskal–Wallis test for all groups), indicating an increasing trend with cervical lesion progression. The diversity composition of ASCUS was similar with LGSIL, while ASCH was closer to HGSIL individuals. The SCC group exhibited the highest diversity compared to NILM (*p* = 0.0019, Kruskal–Wallis pairwise test). Furthermore, the HPV-positive group showed significantly higher diversity compared to HPV-negative individuals (*p* = 0.0242, Kruskal–Wallis pairwise test), but no differences were observed between the single and co-infection groups in comparison to the HPV-negative group.

Faith’s analysis for phylogenetic diversity (PD) showed a significant difference regarding phylogenetic diversity between NILM and ASCH, LGSIL (*p* = 0.071 and *p* = 0.0209, respectively; Kruskal–Wallis pairwise test). Also, HPV-negative individuals presented a higher PD compared to HPV-positive individuals and those with single-genotype HPV infection.

This suggests that the composition of the cervical microbiota in NILM and HPV-negative individuals consists of bacteria that are more phylogenetically distant, albeit present in low abundance (Supplementary Materials—Figure S1).

**Figure 7:**
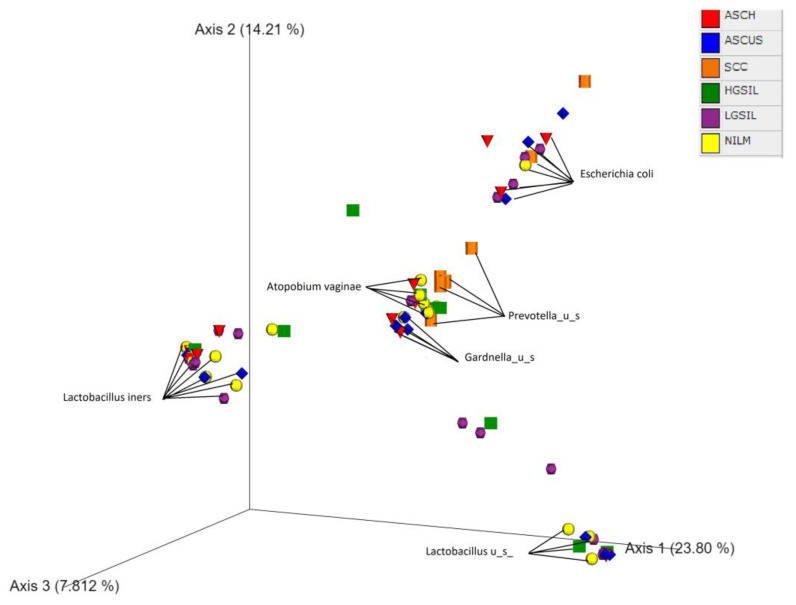
Principal coordinates analysis (PCoA) of microbiota data based on Bray–Curtis distance. PCoA plot with the samples’ colors according to cytology diagnostics.

β-diversity was performed using the PCoA, which is based on the Bray–Curtis index. This analysis demonstrated that the samples were mainly separated into modest proportions of either *Lactobacillus_u_s* and *L. iners* and non-Lactobacillus-dominated categories (Figure 7).

No distinct separation was observed among NILM and different cervical precursor lesions groups. However, a cluster of SCC individuals can be observed exhibiting *Prevotella_u_s* as a predominant genus. ASCUS and ASCH were clustered, pointing to the presence of *Gardnerella_u_s* as a notable genus in these cases. No cluster separation was observed between HPV-negative individuals and those with HPV-positive status, whether it was single or co-infection.

### 3.6. Identification of Potential Biomarkers for the Cervicovaginal Microbiota

We conducted an extended investigation to identify potential bacterial biomarkers associated with cervical lesion progression and HPV infection. To accomplish this, we compared the relative abundance of various bacteria among women with cervical cytological abnormalities and a control group.

**Figure 8:**
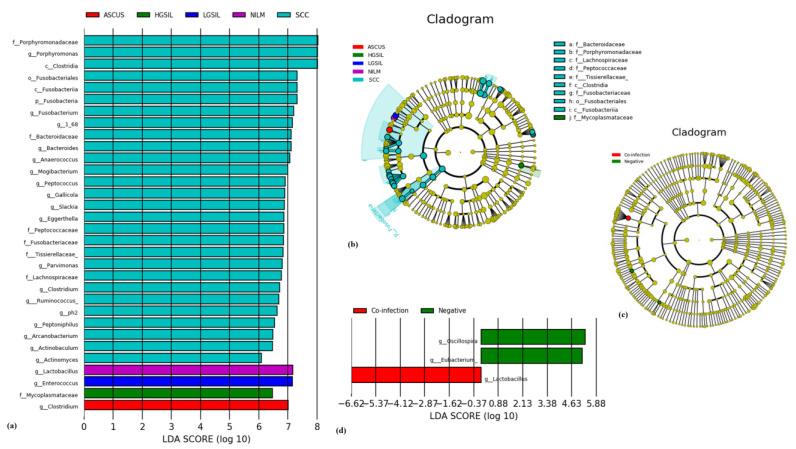
(**a**,**b**) LEfSe results for enriched taxa of cervix in control group and with different cervical lesion and HPV infection status. Differentially expressed taxa are presented as log10 LDA score for cytology classification. (**b**) Differentially expressed taxa cladogram graph. Cladograms represent the phylogenetic relation between microorganisms detected in the different cytology groups (**c**) and related to HPV infection (**d**).

An LEfSe modeling approach was employed, revealing 33 differentially expressed taxa (Figure 8). These bacteria, with a significance level of *p* < 0.05, displayed phylogenetic differences across the various disease stages. Within the SCC group, 28 taxa were identified, with the most abundant being *g_Porphyromonas*, *c_Clostridia*, *g_Fusobacterium*, *g_Bacteroides*, *g_Anaeroccocus*, and *g_Mogibacterium*. The other groups were characterized by a single taxon: NILM-, *g_Lactobacillus*; LGSIL-, *g_Enteroccocus*; HGSIL-, *f_Mycoplamataceae*; and ASCUS-, *g_Costridium*.

The LEfSe analysis revealed significant differences in the microbiota composition at the genus level. Specifically, *g_Lactobacillus* was found to be highly enriched in the HPV co-infection group, whereas the control group showed dominance of the *Oscillospira* and *Eubacterium* genera.

### 3.7. Function of the Cervical Microbiome

This analysis step integrates LEfSe and PICRUSt to identify the functions of microbiota components that are significant in the investigated groups, based on the KEGG database.

The microbiome functions of bacteria in NILM and LGSIL (*p* < 0.05) were mainly associated with membrane transporters, biosynthesis, and other metabolic pathways associated with nutrient acquisition. The most important pathways were ABC transporters, aminoacyl_tRNA biosynthesis (NILM), and purine metabolism (LGSIL).

As the precursor lesion progressed, the microbiota remained partially associated with metabolic functions. For example, in ASCUS, pathways related to galactose and starch/sucrose metabolism were identified, while in ASCH, the nucleotide metabolism pathway was prominent. Furthermore, in HGSIL, pathways associated with DNA replication/repair and antibiotic resistance were observed.

The predicted function of the bacterial microbiota in cancer samples (*p* < 0.05) was primarily associated with the degradation of chemical compounds, synthesis of amino acids and B-group vitamins, and flagellum assembly and motility, as well as pathways related to prostate and bladder cancer (Supplementary Materials—Figure S2).

The function of the microbiota is related to HPV infection status. While HPV-negative samples presented pathways associated with metabolic functions (drug/lipid metabolism), HPV-positive samples exhibited more diverse pathways. In samples with single-HPV-genotype infection, the microbiome function was characterized by pathways related to bacterial motility, flagellar assembly, and the synthesis of bioproducts. HPV co-infection samples presented pathways associated with chromosomes, mismatched repair, and translation factors (Supplementary Materials—Figure S3).

## 4. Discussion

This study is the first conducted in Romania to assess alterations in the cervical microbiota related to different cytological classes and HPV infection. The results of our study revealed an elevated diversity of bacterial species, accompanied by a reduction in *Lactobacillus* spp., which indicates changes associated with the progression of lesions. The results presented match the results that Piyathilake et al. reported, which shows that the changes in the microbiota that were seen as the lesions became worse were similar [30].

*L.iners* was found to be the most predominant species across the samples, along with *Lactobacillus_u_s,* independent of cytological status. A notable aspect of our study was the absence of *L. crispatus* in the studied samples. This finding contrasts with the study conducted by Di Paola et al., where *L. crispatus* was identified as the predominant *Lactobacillus* species in Italian female patients who exhibited better clearance of HPV infection [31].

Regarding alpha diversity patterns, the ASCUS samples exhibited a higher similarity to the LGSIL samples, indicating a closer relationship between these two groups. On the other hand, the ASCH samples demonstrated similarities to the HGSIL samples, suggesting a resemblance between them. The diversity pattern can be used as a potential marker to aid in the classification and risk stratification of patients with ASCUS and ASCH lesions in either low-grade or high-grade cervical lesions.

LGSIL samples exhibited a modified microbiota composition, featuring the presence of *E. faecalis*, *E. coli*, and *Gardnerella_u_s*. In the HGSIL patient group, there was a notable increase in the abundance of *Gardnerella_u_s* and *E. coli*. The SCC samples showed increased diversity; the microbiota included *E. faecalis*., *Prevotella* spp., and *A. vaginae*. To note, a few intraepithelial neoplasia lesion samples were presented by *Sneathia* spp.

According to the study conducted by Oh et al., it was proposed that the lack of *L. crispatus* and the predominance of *A. vaginae,* along with the secondary presence of *G. vaginalis* and *L. iners*, were associated with an approximately six-fold increase in the risk of developing cervical LGSIL/HGSIL [32].

Vaginal microbiota serves as the primary line of defense against infections, with its composition playing a crucial role. The potential of *Lactobacillus* sp. such as *L. crispatus, L. gasseri*, and *L. jensenii* to produce lactic acid and hydrogen peroxide (H_2_O_2_) enables them to inhibit the progression of viral and bacterial infections [33].

Our study has also brought attention to another significant finding, which is the correlation between *L. iners* and HPV infection, irrespective of the cervical lesion’s severity. *L. iners* is more abundant in the NILM group, who have either a single HPV infection or HPV co-infection. Furthermore, we noticed an increased abundance of *L. iners* that can be observed in patients with HPV16 single infection compared to those with HPV16 co-infection. *L. iners* has been identified as a transitional species that may contribute to dysbiosis. Several studies have shown an inverse relationship between HPV infection and CIN with dominant *Lactobacillus* spp., except for *L. iners*, which shows an opposite trend, correlated with a higher frequency of HIV, HPV, and HSV-2 infections [34]. In addition, our findings revealed that the patients presenting HPV16 co-infection exhibit low abundances of *L. psittaci* and *L. zeae*.

In our study, we observed that *Gardnerella_u_s* was predominantly present in the HGSIL group, followed by HPV-positive NILM and those with SCC. This suggests that certain bacteria, such as *Gardnerella*, could potentially serve as biomarkers for detecting cervical changes and identifying women at a higher risk of developing persistent HPV infection with the potential to progress to cancer [35]. In the context of persistent hrHPV infection, the presence of *Gardnerella* is mediated by the increase in diversity of cervicovaginal bacteria, which directly precedes the progression to dysplasia. *Gardnerella vaginalis* and *Atopobium vaginae* have also been proposed as molecular markers, as both could be involved in biofilm formation that may contribute to virus persistence [36].

*Prevotela_u_s* was most abundant in the SCC and HGSIL samples. It was also found that samples with HPV co-infection presented an increased abundance. This finding is noteworthy considering the research conducted by Castañeda-Corzo et al., which demonstrated the potential role of *Porphyromonas* and *Prevotella* in the development of oral cancers [37]. Moreover, *Porphyromonas* and *Prevotella* have been identified as marker genera associated with cervical cancer [38]. Another study has also provided support for the higher abundance of *Prevotella* and *Gardnerella* in women with persistent hrHPV infection and HGSIL [39].

Based on the proposed mechanisms of chronic inflammation, anti-apoptotic activity, and production of carcinogenic substances, it can be expected that *Prevotella* may play a crucial role in the development of cervical cancer within the context of HPV persistence [40].

Consistent with previous research, HPV16 continues to be the most prevalent genotype when considering the distribution of HPV genotypes [41]. Our findings showed that the HPV16 co-infection group exhibited an increased abundance of *Prevotella_u_s*, while the HPV16 single-infection group showed an increased abundance of *Gardnerella_u_s* and *E. coli*; therefore, we can hypothesize that a specific microbiota composition in this region may contribute to the persistence of HPV infection. These observations suggest that the presence and abundance of certain bacterial species could potentially influence the outcome of HPV infection and its progression.

Moreover, previous studies have revealed an association between the presence of *Sneathia* spp. and HPV detection and/or the development of cervical neoplasms. It was shown that a higher prevalence of *Sneathia* was found in patients who are HPV-positive and have dysplastic precursor lesions [5,31,42]. Furthermore, the link to HPV positivity indicated *Sneathia* spp. as a potential biomarker for viral status [18].

In contrast to the studies already mentioned, our study detected *Sneathia* spp. only in four samples with diverse cytology results (HPV-positive), and its abundance was found to be very low. Interestingly, Liu et al. reported a decrease in the population of *Sneathia* spp. in the HPV infection groups, which contradicts the findings of previous studies that identified it as a risk factor [43].

The pathogenic mechanisms underlying the interaction between the microbiota and HPV are still not fully understood, and it is important to identify the most effective therapeutic strategies. The analysis of the microbiota in clinical practice among HPV-positive patients holds promise in identifying women who are at an increased risk of HPV progression or persistence [44].

Besides identifying alterations in the taxonomic landscape during the progression of cervical cancer, we also evaluated the functional composition of the microbiome.

The NILM group was mainly characterized by ABC transporters and aminoacyl-tRNA biosynthesis pathways. Intestinal prokaryotes and archaea acquire amino acids via ABC transporters. These amino acids serve as building blocks for the synthesis of microbial proteins, including the production of aminoacyl-tRNA. Additionally, they contribute to microbial energy metabolism and can be converted into various physiologically active substances, such as neurotransmitters and hormones [45]. The benzoate degradation pathway exhibited a lower LDA score in our data analysis. This pathway was specifically associated with *L. crispatus*, suggesting that the anaerobic biodegradation of benzoate may be linked to the positive effects observed in the vaginal microbiota of healthy women [46]. The transporter pathway was also found to be enriched in the LGSIL group. Within the ASCUS group, we observed a notable enrichment of the starch and sucrose metabolism pathway. These findings are similar to those of other studies that demonstrated an enrichment of this pathway in healthy individuals compared to those with cervical cancer [47], prostate cancer [48], and bladder cancer [49].

Among the significantly modified KEGG pathways in the SCC group, porphyrin and chlorophyll metabolism were noted. Liu et al. found the same pathway affected by HPV infection in cervical lesions [43]. Bacterial motility proteins and flagellar assembly were also found to be enriched in the SCC group and the HPV-positive group with a single genotype. These findings are consistent with the results obtained by Oniwera et al., who suggested that these pathways are associated with a diverse microbiota and are important for enhancing bacterial survival and potentially contributing to dysbiosis [50]. Moreover, B-group vitamin synthesis was significantly abundant in the SCC group. The folate metabolism pathway has been reported to be essential in proliferating tissues [51] and the fecal microbiome of prostate cancer patients [48] and cervical cancer patients [47]. Therefore, enrichment of the folate biosynthesis pathway can be considered to be involved in oncogenesis. HPV co-infected samples exhibited an enrichment of the mismatch repair pathway. It has been documented that enteropathogenic *E. coli* can decrease MSH2 and MLH1 protein levels, which are vital for the mismatch repair process [52]. Additionally, the human gastric pathogen *Helicobacter pylori* can suppress the expression of mismatch repair genes, partly through the modulation of miRNAs [53].

These results suggest that as precursor lesions become worse, the functional profile of the microbiota changes and becomes linked to certain metabolic pathways and processes. The microbiota in cancer samples may play a role in various biological processes and potentially contribute to the development or progression of cancer. The presence of HPV and the specific infection status can influence the functional profile of the microbiota.

In addition, having a comprehensive understanding of the microbiota could facilitate a targeted and personalized therapeutic approach involving the use of antibiotics and probiotics.

Even though more clinical studies are needed to fully understand the link between the microbiota and the progression of cervical lesions in high-risk patients, using antibiotics and probiotics at the same time may be a good way to change the microbiota. This approach has the potential to prevent the persistence and progression of HPV-related lesions towards cervical cancer [54].

Nevertheless, it is important to acknowledge the limitations of the current study. Due to the small number of patients included in this study, the findings may not be representative of the entire Romanian female population since the samples were collected from a specific geographical region located in the north-east of the country. Given the high incidence of cervical cancer in Romania, conducting this study within our population was deemed essential. However, further research is warranted to determine if the observed microbiota pattern is consistent across broader regions and populations within Romania.

The identification of these markers has the potential to serve as a foundation for future therapeutic strategies, including the targeted administration of probiotics. Bacterial markers derived from the cervicovaginal microbiota could be employed as a predictive model for conducting further research on larger sample sizes. This research could focus on identifying appropriate probiotics for women with persistent HPV infection, precancerous lesions at various stages, or invasive cancer. Implementing such strategies could potentially impact the outcomes of chemotherapy and radiotherapy.

Our study revealed a distinct pattern within the studied group when considering the Lactobacillus species, which differs from findings reported in other populations.

The presence of *L. iners* and the absence of *L. crispatus* together with *Atopobium* spp., *Prevotella* spp., and *Gardnerella* spp. could define the landscape for severe cervical lesions. The potential use of specific bacterial species as biological markers is intriguing from a clinical standpoint since women with a particular composition of the cervical microbiota may require more frequent monitoring or alternative therapeutic approaches.

The present findings suggest that women included in this study may exhibit a deficiency of protective *Lactobacillus* spp. or that their cervical microenvironment may not be suitable for their growth. Notably, the distinctiveness of our study, compared to the existing literature, lies in the almost undetectable presence of *L. crispatus*, even among the HPV-negative NILM group. Instead, *L. iners* predominates in our studied groups, giving them a greater vulnerability to the persistence of HPV and the progression of lesions to invasive cancer.

The high incidence of cervical cancer in Romania cannot be solely attributed to inadequate screening. Thus, these particularities of the microbiota could justify the persistence of HPV infection and the progression of cervical lesions.

## 5. Conclusions

In conclusion, the composition of the cervical microbiota appears to be specific to populations and even individuals.

The current research demonstrated a higher microbiota diversity in patients with HGSIL and SCC diagnosis compared to NILM individuals. This study also revealed the absence of *L. crispatus* and the presence of *L. iners*, even in the NILM HPV-negative group. In HGSIL patients, a small number of cases showed the presence of *Sneathia* spp. with relatively low abundances, while their microbiota was predominantly dominated by *Gardnerella* spp. and *E. coli*.

Gaining a better understanding of the composition of the cervical microbiota holds the potential to revolutionize personalized diagnosis and the management of cervical dysplasia and cancer.

By delving into the intricacies of the microbiota, we can unlock valuable insights that enable tailored approaches to diagnosis and treatment, ultimately improving outcomes for patients.

## Figures and Tables

**Table 1:** The demographic characteristics and reproductive/sexual-lifestyle-related risk factors of the studied groups.

	Total Number of Cases, *n* = 85 (100%)							
	NILM HPV-	NILM HPV+	ASCUS HPV+	LGSIL HPV+	ASCH HPV+	HGSIL HPV+	Cancer HPV+	*p*-Value
**Number of cases**	*n* = 11	*n* = 9	*n* = 16	*n* = 18	*n* = 13	*n* = 9	*n* = 9	
(% from total number of cases)	(12.79%)	(10.46%)	(18.82%)	(21.17%)	(15.29%)	(10.46%)	(10.46%)	
**Age**								<0.0001 *
Mean ± SD	38.27 ± 9.166	36.78 ± 7.823	37.54 ± 10.080	33.94 ± 9.175	33.88 ± 8.484	38.00 ± 7.984	63.20 ± 12.260	
**BMI**								0.6970
<18.5	0	1 (11.1%)	1 (6.25%)	2 (11.1%)	1 (7.7%)	0	1 (11.1%)	
18.5–25	6 (54.5%)	7 (77.8%)	12 (75%)	12 (66.6%)	8 (61.5%)	3 (33.3%)	4 (44.4%)	
25–30	3 (27.3%)	0	3 (18.75%)	2 (11.1%)	2 (15.4%)	4 (44.4%)	2 (22.2%)	
>30	2 (18.2%)	1 (11.1%)	0	2 (11.1%)	2 (15.4%)	2 (22.2%)	2 (22.2%)	
**Smoking**								0.3356
Yes	1 (9.1%)	3 (33.3%)	7 (43.75%)	4 (22.2%)	2 (15.4%)	3 (33.3%)	1 (11.1%)	
No	10 (98.9%)	6 (66.7%)	9 (56.25%)	14 (77.7%)	11 (84.6%)	6 (66.6%)	8 (88.8%)	
**Educational level**								**0.0021**
Elementary school	2 (18.2%)	0	0	0	1 (7.7%)	2 (22.2%)	5 (55.5%)	
Professional school	1 (9.1%)	1 (11.1%)	0	0	0	0	1 (11.1%)	
High school	4 (36.35%)	3 (33.3%)	7 (43.75%)	6 (33.3%)	9 (69.3%)	5 (55.5%)	3 (33.3%)	
Bachelor	4 (36.35%)	5 (55.6%)	9 (56.25%)	12 (66.6%)	3 (23.1%)	2 (22.2%)	0	
**Living area**								**0.0177**
Urban	8 (72.7%)	8 (88.9%)	14 (87.5%)	17 (94.4%)	9 (69.3%)	7 (77.7%)	3 (33.3%)	
Rural	3 (27.3%)	1 (11.1%)	2 (12.5%)	1 (5.6%)	4 (30.7%)	2 (22.2%)	6 (66.6%)	
**Marital status**								**<0.0001**
Married	9 (81.8%)	9 (100%)	11 (68.75%)	12 (66.6%)	11 (84.6%)	9 (100%)	5 (55.5%)	
Not married	1 (9.1%)	0	5 (31.25%)	6 (33.3%)	2 (15.4%)	0	0	
Widowed	1 (9.1%)	0	0	0	0	0	4 (44.4%)	
**Age of first sexual life**								0.3098
<20 years old	7 (63.6%)	5 (55.6%)	15 (93.75%)	14 (77.7%)	10 (76.9%)	8 (88.8%)	6 (66.6%)	
≥20 years old	4 (36.4%)	4 (44.4%)	1 (6.25%)	4 (22.2%)	3 (23.1%)	1 (11.1%)	3 (33.3%)	
**Number of sex partners**								0.2081
1	5 (45.4%)	4 (44.4%)	5 (31.25%)	7 (38.9%)	5 (38.5%)	3 (33.3%)	7 (77.7%)	
2	1 (9.10%)	4 (44.4%)	4 (25%)	6 (33.3%)	2 (15.4%)	1 (11.1%)	2 (22.2%)	
≥3	6 (54.5%)	1 (11.2%)	7 (43.75%)	5 (27.7%)	6 (46.1%)	5 (55.5%)	0	
**Sexual frequency**								**<0.0001**
1 time per week	3 (27.3%)	1 (11.1%)	1 (6.25%)	5 (27.7%)	7 (38.8%)	1 (11.1%)	0	
2–3 times a week	6 (54.5%)	8 (88.9%)	13 (81.25%)	11 (61.1%)	3 (23.1%)	6 (66.6%)	0	
≥4 times a week	1 (9.1%)	0	0	0	2 (15.4%)	1 (11.1%)	0	
no	1 (9.1%)	0	2 (12.5%)	2 (11.1%)	1 (7.7%)	1 (11.1%)	9 (100%)	
**Pregnancies**								0.1531
≤1	4 (36.4%)	3 (33.3%)	9 (56.25%)	13 (70.6%)	7 (54.5%)	3 (33.3%)	2 (22.2%)	
≥2	7 (63.6%)	6 (66.7%)	7 (43.75%)	5 (29.4%)	5 (45.5%)	6 (66.7%)	7 (77.7%)	
**Abortions**								0.2577
≤1	10 (90.9%)	7 (77.8%)	14 (87.5%)	17 (94.1%)	11 (81.8%)	5 (55.6%)	7 (77.7%)	
≥2	1 (9.1%)	2 (22.2%)	2 (12.5%)	1 (5.9%)	2 (18.2%)	4 (44.4%)	2 (22.2%)	
**Births**								0.5047
≤1	5 (45.5%)	3 (33.3%)	10 (62.5%)	13 (70.6%)	9 (72.7%)	5 (55.6%)	5 (55.5%)	
≥2	6 (54.5%)	6 (66.7%)	6 (37.5%)	5 (29.4%)	4 (27.3%)	4 (44.4%)	4 (44.4%)	

* *p*-value obtained using ANOVA test.

## Data Availability

The authors confirm that the data supporting the findings of this study are available within the article and its Supplementary Materials.

## Supplementary Materials

The following supporting information can be downloaded at https://www.mdpi.com/article/10.3390/jcm12154979/s1, Figure S1: Comparison of the non-parametric Shannon, Faith’s, and OTU’s diversity indices for healthy controls and patients with different cytologies (a–c), HPV-negative versus -positive patients (d–f) and HPV-negative versus single and co-infection HPV-positive cases (g,h); Figure S2: The analysis of microbial function and metabolism based on the KEGG database related to cytological status; Figure S3: The analysis of microbial function and metabolism based on the KEGG database related to HPV infection status. Table S1. Correlation between Pap smear and biopsy results.
