# Supplementary material for: Exploring Microbiota Diversity in Cervical Lesion Progression and HPV Infection through 16S rRNA Gene Metagenomic Sequencing
Source: J Clin Med. 2023 Jul 28;12(15):4979. doi: 10.3390/jcm12154979 (PMC10420036; doi:10.3390/jcm12154979)
Supplement: Supplementary file 1 [file jcm-12-04979-s001.zip › jcm-2531359-supplementary/Supplementary/Table S1.pdf]

Table S1. Correlation between Pap smear and biopsy results.

PAP SMEAR RESULT	*HPV STATUS*	*BIOPSY POUNCHED*	*CASES n=85*
NILM	HPV -	-	11
	HPV HR +	-	9
ASCUS	HPV HR +	CIN1	13
	HPV HR +	cervicitis	4
LSIL	HPV HR +	CIN1	16
	HPV HR +	CIN2	2
ASCH	HPV HR+	CIN1	2
	HPV HR +	CIN2	7
	HPV HR +	CIN3	4
HSIL	HPV HR+	CIN2	7
	HPV HR+	CIN3	3
SCC	HPV HR +	carcinoma	9
