# Supplementary figures and images for: Exploring Microbiota Diversity in Cervical Lesion Progression and HPV Infection through 16S rRNA Gene Metagenomic Sequencing
Source: J Clin Med. 2023 Jul 28;12(15):4979. doi: 10.3390/jcm12154979 (PMC10420036; doi:10.3390/jcm12154979)

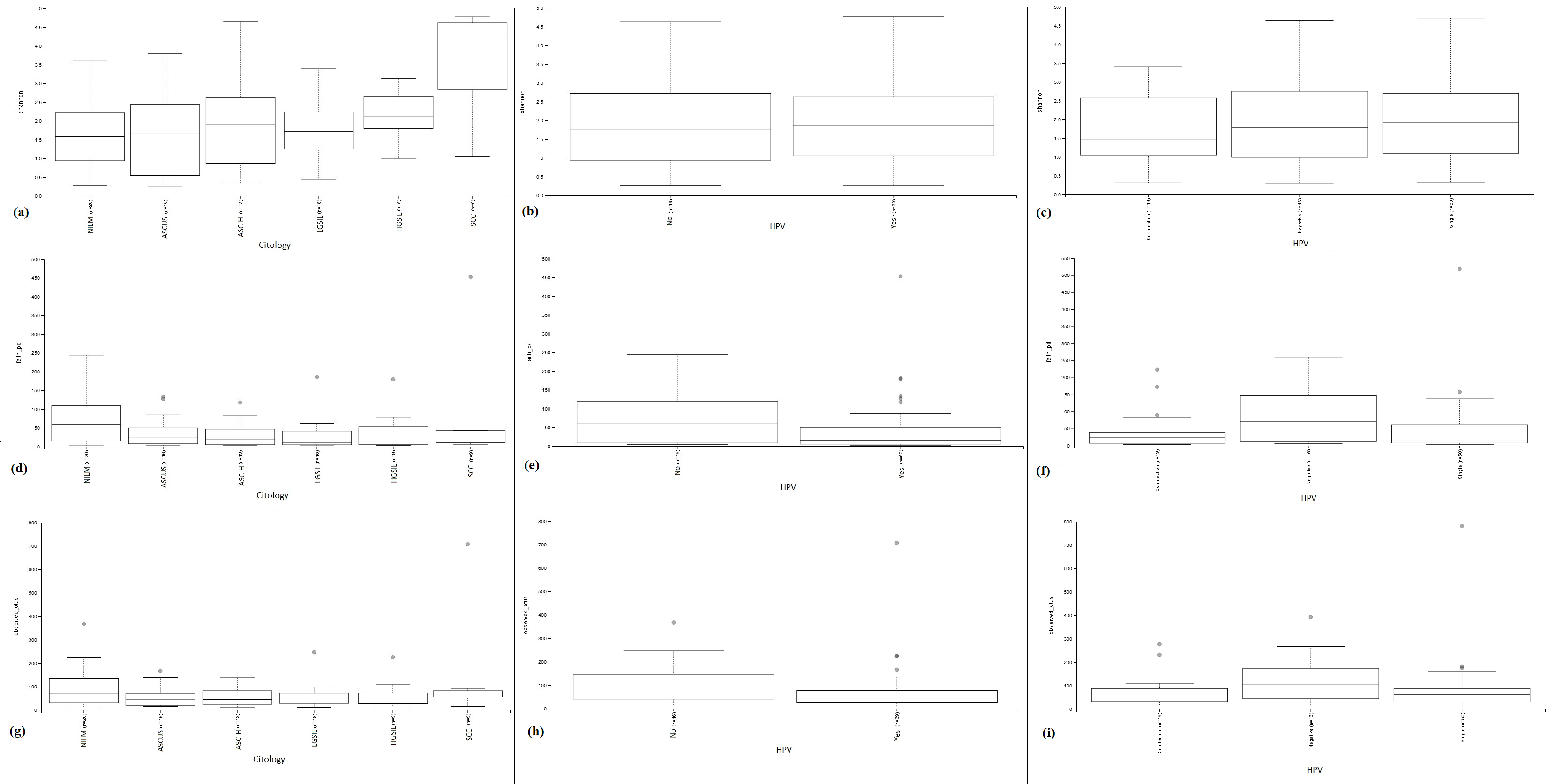

Supplement: Supplementary file 1 [file jcm-12-04979-s001.zip › jcm-2531359-supplementary/Supplementary/Figure S1.tif]

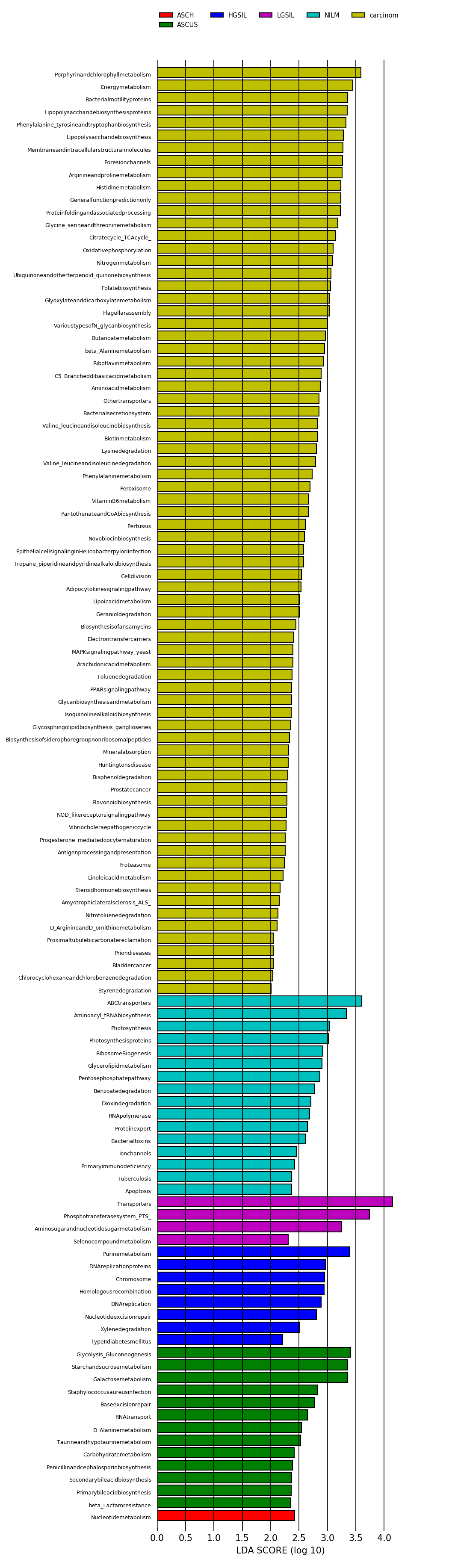

Supplement: Supplementary file 1 [file jcm-12-04979-s001.zip › jcm-2531359-supplementary/Supplementary/Figure S2.tif]

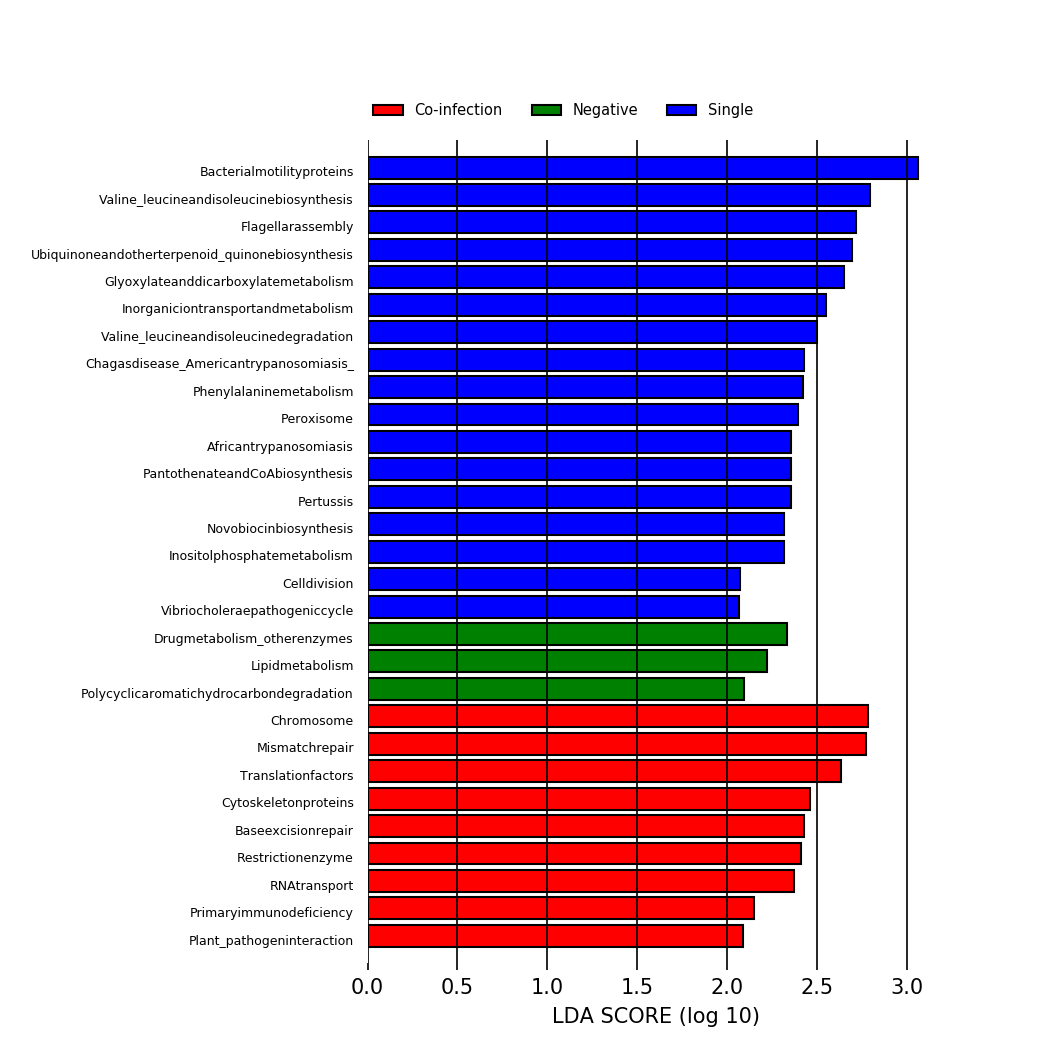

Supplement: Supplementary file 1 [file jcm-12-04979-s001.zip › jcm-2531359-supplementary/Supplementary/Figure S3.tif]
